# Supplementary material for: SyNDI: synchronous network data integration framework
Source: BMC Bioinformatics. 2018 Nov 6;19:403. doi: 10.1186/s12859-018-2426-5 (PMC6219086; doi:10.1186/s12859-018-2426-5)
Supplement: Supplementary file 5 — Files of the example presented in Additional file 4. This additional file is a zip package that contains all files used in Additional file 4. Detailed descriptions of each of these files are presented in a README file included in this zip package. (GZ 30411 kb) [file 12859_2018_2426_MOESM5_ESM.gz › SyNDITools/Meme2Fimo/meme_4.9.0_4.tar.gz!/meme_4.9.0/doc/uniprobe2meme.html]

uniprobe2meme


# uniprobe2meme

**Usage:** `uniprobe2meme [options]`

**Description:**

Read a UNIPROBE matrix file and convert to MEME format. You may concatenate
files for more than one motif.

**Input:**

Reads `standard input`.

Concatenated UNIPROBE files describe one or more motifs. Each motif
has a header line and a position-weight matrix.

A log-odds matrix and a probability matrix are output for each
motif. The probability matrix is computed using
pseudo-counts consisting of the background frequency (see -bg, below)
multiplied by the total pseudocounts (see -pseudo, below). The log-odds
matrix uses the background frequencies in the denominator and is log
base 2.

**Options:**

-skip <ID>
:   skip this ID (may be repeated)

-numseqs <numseqs>
:   assume frequencies based on this many sequences; default: 20

-truncate\_names
:   truncate motif names at first underscore

-numbers
:   use numbers instead of strings as motif names

-bg <background file>
:   file with background frequencies of letters; default: uniform background

-pseudo <total pseudocounts>
:   add <total pseudocounts> times letter background to each frequency; default: 0

-logodds
:   print log-odds matrix, too; default: print frequency matrix only

-url <website>
:   website for the motif; The untruncated ID is substituted for MOTIF\_NAME; default: no url

-h
:   print usage message

**Output:**

Writes MEME format to standard output.

**Sample Input:**

```
Arid3a_primary
A:	0.179645793597971	0.210100830864919	0.18297957170479	0.218158426414591	0.0870705802557497	0.0435293344848582	0.695645391274696	0.886231513936226	0.105526350481291	0.112302355076614	0.815030478488063	0.719906706101697	0.598597229834128	0.469818137392637	0.233954380973979	0.267838631058226	0.240559605564699
C:	0.266648735950844	0.218430924209102	0.213084153545938	0.203797703013696	0.110886816294852	0.0958703394836635	0.0104078225875119	0.00354104133931097	0.00470109424317215	0.181644431061179	0.045569847543415	0.0821358973477016	0.0458429114703258	0.282556547844063	0.229160661497462	0.231747372592299	0.300737862311012
G:	0.382823288901203	0.353259680323954	0.340965693699501	0.18760023292416	0.0821358973477016	0.045569847543415	0.181644431061179	0.00470109424317215	0.00354104133931097	0.0104078225875119	0.0958703394836635	0.110886816294852	0.0853202791343203	0.0573452956112842	0.190290024780233	0.133765504128307	0.201112183210201
T:	0.170882181549982	0.218208564602024	0.262970581049772	0.390443637647554	0.719906706101697	0.815030478488063	0.112302355076614	0.105526350481291	0.886231513936226	0.695645391274696	0.0435293344848582	0.0870705802557497	0.270239579561226	0.190280019152016	0.346594932748325	0.366648492221168	0.257590348914088

Arid5a_primary
A:	0.177322769961677	0.160221841004699	0.852203018833067	0.961248539596473	0.0565398220443113	0.930330589214641	0.019950864688249	0.0411316382396864	0.233918811349109	0.339577406902739	0.285389335391289	0.572142692856743	0.285668980971376	0.342798848206545
C:	0.428313310855555	0.324571292259573	0.0267326703147277	0.00379831572782298	0.00400812936891674	0.0051379987521542	0.0141888286179256	0.0875059354874809	0.027935175632631	0.352987545623934	0.13471914100935	0.0784175907242025	0.184067740053308	0.230459946341397
G:	0.22903578216647	0.0324446387621822	0.073955464568744	0.0101183390736629	0.00787744708407332	0.00713820083753976	0.00678864795483705	0.0390246666857379	0.519604520580082	0.184315929397724	0.268289476918125	0.190434321213762	0.262198874546343	0.153946120466092
T:	0.165328137016298	0.482762227973546	0.0471088462834612	0.0248348056020407	0.931574601502699	0.057393211195665	0.959071658738988	0.832337759587095	0.218541492438178	0.123119118075603	0.311602046681237	0.159005395205293	0.268064404428973	0.272795084985967
```
